# Peroxisomes: A New Hub for Metabolic Engineering in Yeast

**DOI:** 10.3389/fbioe.2021.659431

**Published:** 2021-04-07

**Authors:** Natalja Kulagina, Sébastien Besseau, Nicolas Papon, Vincent Courdavault

**Affiliations:** ^1^Université de Tours, EA2106 “Biomolécules et Biotechnologies Végétales”, Tours, France; ^2^Université d'Angers, EA3142 “Groupe d'Etude des Interactions Hôte-Pathogène”, Angers, France

**Keywords:** peroxisomes, yeast, compartmentalization, metabolic engineering, heterologous production

Metabolic engineering represents a continuously broadening collection of tools and strategies aiming at developing cell factories able to efficiently produce numerous compounds of interest. In this context, natural products of plant origin have long been of particular importance, well-known for their valuable properties exploited in drug, cosmetic, food, and biofuel industries (Cragg and Newman, 2013). Currently, the sourcing of many of these metabolites still relies on the exploitation of natural plant resources, which may promptly cause supply shortages (Courdavault et al., 2020). Over the last 10 years, heterologous production has been revealed as a cost-effective and sustainable alternative to conventional strategies of production, where yeasts, particularly *Saccharomyces cerevisiae*, are widely used as a robust and easy to genetically manipulate platform (Guirimand et al., 2020b).

While many achievements have been reported to date in the field, the expression of exogenous enzymes in yeast often results in poor yields, due to the accumulation of toxic biosynthetic intermediates and/or the formation of undesirable byproducts generated by competing endogenous pathways. To address the analogous issues, as well as to provide the optimal physiochemical conditions for enzymatic reactions, the natural producers of specialized metabolites commonly use intracellular compartmentalization of their biosynthetic pathways to insulate the enzymes into specific organelles. For instance, in the Madagascar periwinkle (*Catharanthus roseus*), the monoterpene indole alkaloid (MIA) pathway biosynthetizing the prominent cytotoxic compounds vincristine/vinblastine displays a highly sophisticated cellular and subcellular compartmentalization, with at least three cell types and five distinct subcellular compartments, which directly contributes to the tight regulation of the pathway (Courdavault et al., 2014; Guirimand et al., 2020a). Other organisms adopt a simpler compartmentalization, such as the mold *Penicillium chrysogenum*, where the two last reactions of the penicillin pathway take place in the peroxisome (Kistler and Broz, 2015), the importance of which was demonstrated in penicillin-producing yeast *Hansenula polymorpha* (Gidijala et al., 2009). Thus, the artificial relocalization of heterologous enzymes to specific organelles of the host cells emerged as an alternative approach to limit toxicity and hijacking issues (Huttanus and Feng, 2017). While several organelles have been assayed, such as the mitochondria (Szczebara et al., 2003) and ER (Thodey et al., 2014), the peroxisome progressively stood out as an appealing candidate due to its intrinsic advantageous specificities, including the highest protein content compared with that of other organelles (Sibirny, 2016), which could be an attractive feature for protein overexpression.

The peroxisome is a single-layer DNA-free organelle, involved in the β-oxidation of fatty acids, which provides an endogenous acetyl-CoA pool—an important cofactor for numerous enzymes. It also contains hydrogen peroxide-metabolizing oxidases and catalases and thus participates in hydrogen peroxide signaling and cell detoxification (Hammer and Avalos, 2017). Moreover, peroxisome hosts biosynthetic pathways/enzymes, including the last steps of the mevalonate (MVA) pathway (Kovacs et al., 2007; Simkin et al., 2011). Peroxisome assembly mostly relies on cytosol-born peroxisome matrix proteins, which are posttranslationally targeted to peroxisomes via peroxisomal targeting sequences (more common C-terminal PTS1 and N-terminal PTS2) (Rucktäschel et al., 2011), and biogenesis-related peroxisome proteins peroxins. Most of the peroxins are involved in the import of PTS1/PTS2-containing proteins, which are recognized by the specific receptor-like peroxins (Pex5 and Pex7/Pex18, Pex21, respectively), further bound to the membrane peroxin docking complex (Pex13, Pex14, and Pex17), and translocated into the peroxisome, where the protein cargo is released and the receptors are recycled back to the cytosol mediated by membrane Pex2, Pex10, and Pex12 complex (Rucktäschel et al., 2011; Sibirny, 2016). As for metabolite trafficking, initial *in vitro* assays suggested membrane permeability to metabolites of a wide size range, which could not be confirmed *in vivo*. Subsequently several specific transporters have been identified, such as Ant1p for the import of ATP/AMP (Antonenkov and Hiltunen, 2012) and Pxa1/Pxa2 for fatty acids (Nyathi et al., 2010), and metabolite transport was finally shown *in vivo* to be size dependent in a pore-based (diameter of 0.57–0.65 nm) manner with free diffusion of small molecules and more selective trafficking of larger compounds (DeLoache et al., 2016).

The identification of peroxisomal-targeting peptides indeed enabled the efficient targeting of heterologous enzymes to peroxisomes by basically adding the three-amino acid PTS1 at the extreme C-terminal protein end. For instance, this strategy was successfully applied for targeting three lycopene pathway enzymes into the yeast *Pichia pastoris* peroxisomes resulting in a boost in lycopene production (Lee et al., 2009). Moreover, given the peroxisome intrinsic β-oxidation and fatty acid metabolism, peroxisomal targeting has been rationally used to improve heterologous production of fatty acid-derived compounds, such as biofuels (Sheng et al., 2016; Zhou et al., 2016), which is reviewed in Hammer and Avalos (2017) in more detail. Beyond these pioneering examples, peroxisomes have been very recently exploited as a reliable hub for metabolic engineering and heterologous production of other valuable compounds. In this opinion article, we discuss the outstanding and recent publications redirecting enzymes or pathways to peroxisomes for the production of alkaloids (Grewal et al., 2021), monoterpenoids (Dusséaux et al., 2020; Gerke et al., 2020), and squalene (Liu et al., 2020). These benchmark articles also nicely shed light on peroxisome intrinsic properties/capacities aiming at further amplifying their potential in the near future (Figure 1).

**Figure 1:**
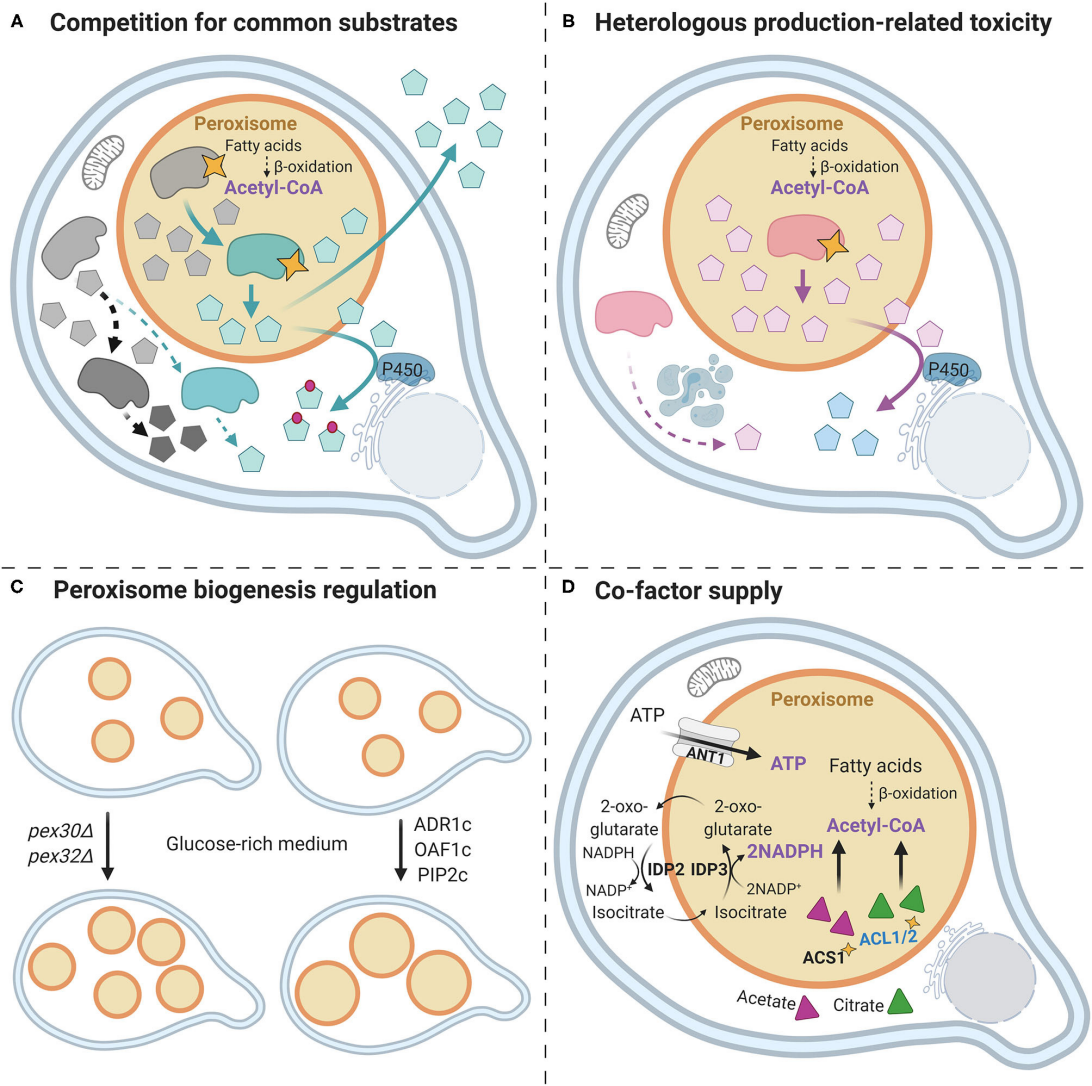
Strategies for using yeast peroxisome as an efficient microfactory. **(A)** Competition for common substrates. Addressing native/heterologous enzymes to the *Saccharomyces cerevisiae* peroxisomes prevents the competition for common substrates between heterologous and native metabolic pathways, which results in improved yield (Dusséaux et al., 2020) [geranyl pyrophosphate (GPP)-derived compounds]. Gray forms represent native enzymes (dark gray—the competing enzyme, light gray—common substrate-supplying enzyme), and black dotted arrows show the native metabolic pathways. Green forms indicate the heterologous enzymes, and green arrows show heterologous biosynthetic pathways. Yellow stars represent the peroxisomal targeting via the addition of PTS1/ePTS1 signal. The light blue pentagons represent the product/intermediate; the gray pentagons—natively synthesized compounds. In some cases, the dual-localization approach is beneficial for production (squalene production in Liu et al., 2020). In addition, ER proximity to peroxisomes is potentially beneficial for P450 enzymes (Dusséaux et al., 2020) (downstream GPP-derived molecules). **(B)** Heterologous production-related toxicity. The cytotoxicity can be caused by the overexpression of heterologous enzymes (Grewal et al., 2021 (tNCS) or by the synthesized product (Dusséaux et al., 2020, Gerke et al., 2020) (geraniol). Addressing the corresponding enzymes to the peroxisome via adding the PTS1 signal (yellow star) allows to bypass these limitations and enhance heterologous production. The cytotoxic product/intermediate can be then either accumulated in the peroxisome or further converted to a nontoxic product. **(C)** Peroxisome biogenesis regulation. The overexpression of *ADR1, OAF1*, and *PIP2*, engineered in order to be constitutively active in the glucose-rich medium (*ADR1c, OAF1c*, and *PIP2c)*, results in the enhanced size, and thus the capacity of peroxisomes (Grewal et al., 2021) [enhanced (*S*)—norcoclaurine titer]. The simultaneous deletion of *PEX30* and *PEX32* leads to an increased number of peroxisomes (Gerke et al., 2020) (increased geraniol production). **(D)** Peroxisome intrinsic cofactor supply. The yellow star represents PTS1 signal for peroxisomal localization. *IDP2* and *IDP3*, as well as *ACS1* and *ACL1/2* (targeted to the peroxisome, which allows generating acetyl-CoA from acetate and citrate, respectively), were overexpressed to enhance NADPH and acetyl-CoA supply, respectively. *ANT1* was overexpressed to increase ATP import. These optimizations led to an improved squalene production yield (Liu et al., 2020).

First, the group of Kamparis showed that relocating enzymes to yeast peroxisomes (Figures 1A,B) can be exploited for an improved biosynthesis of geranyl pyrophosphate (GPP)-derived molecules, such as monoterpenes, plant cannabinoid precursor cannabigerolic acid (CBGA), and monoterpene indole alkaloids precursor 8-hydroxygeraniol (Dusséaux et al., 2020). These natural products represent an important group of metabolites (or their precursors) with fragrant and pharmacological properties. They derive from a common GPP scaffold that is also used for the synthesis of essential primary metabolites. In yeast metabolic engineering, given the intense competition with endogenous and heterologous pathways, the GPP supply was determined as the main limitation (Ignea et al., 2019). In fact, it challenges the heterologous biosynthesis of downstream compounds, particularly the essential yeast ergosterol, which regulates the fungal plasma membrane fluidity balance. For instance, heterologous production of limonene and geraniol remained hitherto relatively modest: 0.9 g/L (Cheng et al., 2019) and 1.68 g/L (Jiang et al., 2017), respectively. In yeast, GPP is rapidly converted into the precursor of the sterol pathway farnesyl diphosphate (FPP) by the same cytosolic enzyme Erg20p, which also condenses the two precursors of GPP (Ignea et al., 2019). Thus, to bypass the endogenous competition for GPP, the authors targeted the entire GPP biosynthetic pathway, starting from the MVA pathway, into peroxisomes. In addition, the MVA pathway, which supplies the GPP precursors isopentenyl diphosphate (IPP) and dimethylallyl diphosphate (DMAPP), was also optimized via expressing more efficient enzyme variants from the bacterium *Enterococcus faecalis* along with a variant of a GPP-producing enzyme, and different monoterpene synthases such as (*S*)-(–)-limonene or geraniol synthases (Dusséaux et al., 2020). This approach allowed the creation of peroxisomal microfactories that take advantage of the peroxisome endogenous pool of acetyl-CoA to supply the MVA pathway, bypass the competition of the sterol pathway for GPP, and limit cytotoxicity of the resulting product (e.g., geraniol). Remarkably, this led to an up to a 125-fold increase in monoterpene production compared with cytosol-located production, with titers reaching 5.52 g/L of geraniol and 2.58 g/L of (*S*)-(–)-limonene in fed-batch cultivation. Moreover, this peroxisome-based system also improved the downstream monoterpene oxidation catalyzed by cytochrome P450 enzymes, which, for instance, led to a 69-fold improvement of (*S*)-(–)-limonene bioconversion into the menthol precursor *trans*-isopiperitenol. Of note, besides a higher GPP availability, this improvement may also result from a substrate channeling engendered by the proximity of peroxisomes with endoplasmic reticulum where P450s are anchored. On the other hand, to address the cytotoxicity concern and improve the production of geraniol in yeast, the group of Braus not only targeted geraniol-producing enzymes into peroxisomes but also increased the peroxisome number and enhanced yeast tolerance to geraniol (Gerke et al., 2020) (Figures 1B,C). It was previously shown that the amount of yeast peroxisomes is nutrient dependent and is induced by oleate, which promotes β-oxidation of long-chain fatty acids to produce acetyl-coA. This process was found to rely on peroxin Pex30-, Pex31-, and Pex32-related regulation (Vizeacoumar et al., 2004), and the simultaneous deletion of *PEX30* and *PEX32* led to an increase in peroxisome number in a glucose-rich medium (Gerke et al., 2020) (Figure 1C). Interestingly, *PEX* deletion allowed the identification of a concomitant mutation in *BUL1* leading to the synthesis of a truncated α-arrestin-like adaptor protein that resulted in an increased geraniol tolerance without any impact on cell growth. By following these improvements, the authors successively improved geraniol production by 13% in geraniol-sensitive peroxisomal geraniol-producing strain, 63% in geraniol-tolerant cytoplasmic geraniol-producing strain, and up to 80% in geraniol-sensitive peroxisomal geraniol-producing strain. Besides revealing the positive effect of geraniol tolerance improvement of yeast platforms, this study also shed light on the importance of flexibility and modularity of peroxisome populations for metabolic engineering purposes. Overall, these seminal studies thus established the potential of yeast peroxisome as a tool to circumvent endogenous competition for common substrates and to manage product/intermediate toxicity. Undoubtedly, it will streamline engineering approaches of biosynthetic pathways sharing similar limiting factors.

The management of heterologous enzyme toxicity by peroxisomes and the modulation of peroxisome capacity were also pointed out in the work of Dueber's group (Figures 1B,C) regarding (*S*)-norcoclaurine production in yeast (Grewal et al., 2021). (*S*)-norcoclaurine is the common precursor of benzylisoquinoline alkaloids (BIAs), which include important therapeutically active molecules as the anticancer noscapine and the analgesic codeine and morphine (Hagel and Facchini, 2013). This major precursor is synthesized by norcoclaurine synthase (NCS) through the condensation of dopamine and 4-hydroxyphenylacetaldehyde (4-HPAA) (Lichman et al., 2015). The heterologous production of downstream BIAs thus requires a high activity of NCS to offer a sufficient (*S*)-norcoclaurine supply. In this work, investigators identified a more active variant of NCS through truncation of the 20-first N-terminal residues from the medicinal plant *Coptis japonica* NCS (tNCS) thus removing a vacuolar transit peptide. However, it was observed that tNCS exhibited cytotoxicity through a yet unknown mechanism when overexpressed in the yeast cytosol. Therefore, tNCS was targeted to the peroxisomes via the addition of an enhanced PTS1 (LGRGRR linker prior to PTS1 SKL peptide improves peroxisomal targeting and sequestration according to DeLoache et al., 2016), which resulted in reduced cytotoxicity and in turn in 54% increase in (*S*)-norcoclaurine titer. In addition, peroxisome-produced (*S*)-norcoclaurine was shown to be accessible for downstream heterologous enzymes of the BIA pathway overexpressed in the yeast cytosol and converting (*S*)-norcoclaurine to (*S*)-reticuline, yielding a 2.1-fold increase in (*S*)-reticuline titer. However, at higher tNCS expression levels, an incomplete peroxisomal localization was observed along with cytotoxicity, which suggested a limited import capacity of peroxisomes that may require further optimization. In this respect, the oleate-dependent transcription factors Adr1, Oaf1, and Pip2, which are known to be involved in peroxisome proliferation, were engineered to benefit from constitutive activity in the glucose-rich medium. Consequently, their overexpression magnified peroxisome size and import capacity (Figure 1C), which resulted in a 47% improvement in (*S*)-norcoclaurine titer. In sum, this work thus described an additional approach to modulate peroxisome capacity size-wise, complementary to the enhancement of peroxisome number, both of which could be potentially used in synergy.

Finally, the relevance of the engineering of squalene production in yeast through peroxisomal targeting of biosynthetic enzymes and modulation of peroxisomal cofactor pools was also recently underlined by the Wei group (Liu et al., 2020). Terpene production by microbes is commonly challenging as they are not directly secreted, but rather stored intracellularly (Ma et al., 2019). This represents a major drawback, amplified by potential cytotoxicity and retro control on endogenous terpene pathways. In yeast, the intracellular storage of these hydrophobic compounds is thought to take place in the cytoplasmic lipid droplets of limited capacities (Ta et al., 2012; Sitepu et al., 2014). In this work, after establishing the storage of the excess of cytoplasmic squalene into peroxisomes, the authors targeted the entire squalene biosynthetic pathway to yeast peroxisomes through the addition of PTS1, which led to a 68-fold improvement in squalene production compared with the parental strain (650 mg/L). In addition, given that squalene derives from acetyl-coA and implies massive consumption of ATP and NADPH, specific yeast enzymes were overexpressed in the yeast chassis (Figure 1D). These include peroxisomal ATP transporter encoding Ant1, Idp2, and Idp3 involved in NADPH generation, as well as peroxisome-targeted Acs1 and *Yarrowia lipolytica* Acl1/2 implicated in the acetyl-CoA synthesis from acetate and citrate, respectively. A coordinated overexpression of these enzymes yielded a substantial increase in the peroxisomal pool of all cofactors. This resulted in an additional 1.65-fold increase in squalene synthesis up to 1.3 g/L. Finally, since multiple compartment storage can dramatically enhance production titers, the authors hybridized cytoplasmic and peroxisomal squalene-overproducing strains to generate a diploid strain that exhibited a 29% increased synthesis of squalene. Consequently, the optimized fed-batch cultivation of the best performing strain yielded a remarkable squalene titer of 11 g/L. Besides the impact of pathway relocation to peroxisomes, these results taught us how the engineering of the peroxisomal cofactor supply confers an additional level of flexibility and adjustment of peroxisome properties leading to increased production titers.

Above all, inspired by natural organizations observed in various organisms, recent advances teach us that relocalization of biosynthetic enzymes or whole biosynthetic pathway to peroxisomes allows insulating toxic enzymes or biosynthetic intermediates. The four outstanding articles highlighted here offer a broadened view of the potential of peroxisomes as a new hub for hosting biosynthetic pathways in metabolic engineering approaches. However, although this cell compartment benefits from an efficient diffusion-type cross-membrane transport of small molecules, which can be further proceeded by downstream cytosolic enzymes, the sequestration of stable toxic intermediates remains delicate. Given that modulating the entire peroxisome trafficking system displays an extensive challenge, acquiring tolerance to toxicity, as for geraniol via *BUL1* mutation, for example, emerges as an appealing alternative. Besides, peroxisome physiochemical conditions may not be suitable/optimal for all non-peroxisomal enzymes in terms of pH or redox state, which may compromise enzyme activity and the relevance of synthetic peroxisomal targeting. Indeed, there are still certain features that require further development, but as a whole, combined with more efficient targeting, increased peroxisome number, optimization of import capacities, cofactor production, and future advances, the peroxisome-based metabolic engineering approaches have the potential to dramatically improve any heterologous production. Thus, peroxisomes can be now considered as a synthetic biology tool, modular, and adaptable to face and resolve further challenges in metabolic engineering.

## Author Contributions

NK, SB, NP, and VC contributed to the writing of this manuscript. All authors contributed to the article and approved the submitted version.

## Conflict of Interest

The authors declare that the research was conducted in the absence of any commercial or financial relationships that could be construed as a potential conflict of interest.
